# LncRNA‐SULT1C2A regulates *Foxo4* in congenital scoliosis by targeting rno‐miR‐466c‐5p through PI3K‐ATK signalling

**DOI:** 10.1111/jcmm.14355

**Published:** 2019-05-02

**Authors:** Chong Chen, Haining Tan, Jiaqi Bi, Lin Li, Tianhua Rong, Youxi Lin, Peiyu Sun, Jinqian Liang, Yang Jiao, Zheng Li, Liang Sun, Jianxiong Shen

**Affiliations:** ^1^ Department of Orthopedic Surgery Peking Union Medical College Hospital, Chinese Academy of Medical Sciences, Peking Union Medical College Beijing China; ^2^ Beijing Zhongke Jingyun Technology Company Ltd. Beijing China; ^3^ Department of Orthopedics Surgery Beijing Hospital of Traditional Chinese Medicine Beijing China

**Keywords:** congenital scoliosis (CS), Foxo4, PI3K‐AKT, rno‐miR‐466c‐5p, somitogenesis, SULT1C2A

## Abstract

Congenital scoliosis (CS) is the result of anomalous vertebrae development, but the pathogenesis of CS remains unclear. Long non‐coding RNAs (lncRNAs) have been implicated in embryo development, but their role in CS remains unknown. In this study, we investigated the role and mechanisms of a specific lncRNA, SULT1C2A, in somitogenesis in a rat model of vitamin A deficiency (VAD)‐induced CS. Bioinformatics analysis and quantitative real‐time PCR (qRT‐PCR) indicated that SULT1C2A expression was down‐regulated in VAD group, accompanied by increased expression of rno‐miR‐466c‐5p but decreased expression of *Foxo4* and somitogenesis‐related genes such as *Pax1*, *Nkx3‐2* and *Sox9* on gestational day (GD) 9. Luciferase reporter and small interfering RNA (siRNA) assays showed that SULT1C2A functioned as a competing endogenous RNA to inhibit rno‐miR‐466c‐5p expression by direct binding, and rno‐miR‐466c‐5p inhibited *Foxo4* expression by binding to its 3′ untranslated region (UTR). The spatiotemporal expression of SULT1C2A, rno‐miR‐466c‐5p and *Foxo4* axis was dynamically altered on GDs 3, 8, 11, 15 and 21 as detected by qRT‐PCR and northern blot analyses, with parallel changes in Protein kinase B (AKT) phosphorylation and PI3K expression. Taken together, our findings indicate that SULT1C2A enhanced *Foxo4* expression by negatively modulating rno‐miR‐466c‐5p expression via the PI3K‐ATK signalling pathway in the rat model of VAD‐CS. Thus, SULT1C2A may be a potential target for treating CS.

## 1 INTRODUCTION

Congenital scoliosis (CS) is a three‐dimensional deformity of the spine caused by congenitally anomalous vertebrae development. The incidence of CS is approximately 0.5 to 1 per 1000 live births,^1^, ^2^ and increasing evidence shows that CS is caused by both genetic abnormalities as well as maternal exposure to medications or toxins.^3^, ^4^ Abnormalities in the developmental milieu during embryo somitogenesis may disrupt the cartilaginous spine, consequently resulting in vertebrae malformations.^5^, ^6^ Vitamin A deficiency (VAD) during pregnancy can induce CS in postnatal rats. In addition, changes in the retinoic acid (RA) pathway can affect somitogenesis,^7^ possibly because RAs control the development of the segmental structure of the vertebrate body during embryogenesis.^8^, ^9^, ^10^ Retinoic acid is a metabolite of vitamin A (retinol) that mediates the critical functions of vitamin A in growth and development. However, the mechanisms underlying VAD‐induced CS remain unclear.

MicroRNAs (miRNAs) are small non‐coding RNAs (ncRNAs) that inhibit or degrade mRNAs by binding to the 3ʹ untranslated region (UTR) of mRNAs. Long non‐coding RNAs (lncRNAs) are non‐protein coding transcripts of >200 nucleotides that modulate many physiological and pathological biological functions via epigenetic regulation and interaction with miRNAs, proteins or mRNAs.^11^ Long non‐coding RNAs can act as competing endogenous RNAs (ceRNAs) to inhibit miRNA activity by sequestering miRNA, thereby reducing the expression of its target genes.^12^, ^13^, ^14^ Recently, lncRNAs were found to play an important role in embryo development.^15^, ^16^ However, the role of lncRNAs in somitogenesis remains largely unclear.

Zhang et al reported that miRNA‐145‐5p (miR‐145) and β‐catenin mRNA (CTNNB1) are overexpressed in adolescent idiopathic scoliosis (AIS) bone tissues and primary osteoblasts, and such aberrant miRNA expression has been shown to influence osteocyte function.^17^ A lncRNA known as lncAIS, for its involvement in the pathogenesis of AIS, has been reported to suppress the osteogenic differentiation of mesenchymal stem cells (MSCs). Specifically, lncAIS impacts the function of NF90 and HOXD8 and hinders RUNX2 transcription.^18^ Previously, we performed sequencing data analysis to build ceRNA networks in embryonic tissues of rats with VAD‐induced CS and found that, based on Kyoto Encyclopedia of Genes and Genomes (KEGG) pathway analysis, a SULT1C2A‐rno‐miR‐466c‐5p‐*Foxo4* axis as well as the Phosphoinositide 3 kinases (PI3K)‐AKT pathway appear to be involved in CS pathogenesis based on bioinformatics prediction and sequence analysis.^19^

In the present study, we aimed to confirm the expression of the lncRNA SULT1C2A in a rat model of VAD‐induced CS and to explore the molecular mechanism of the SULT1C2A‐rno‐miR‐466c‐5p‐*Foxo4* axis in CS. This study of the effect of ceRNA dysregulation within the pathogenesis of VAD‐CS provides insight into the mechanisms of somitogenesis and suggests that SULT1C2A may be a potential target for treating CS.

## 2 MATERIALS AND METHODS

### 2.1 Rat model of VAD‐induced CS

Sprague‐Dawley rats (20 weeks old, weighing 200‐230 g) were obtained from SPF Biotechnology Co., Ltd (Beijing, China). The Institutional Animal Welfare Committee of the Peking Union Medical College Hospital and the Laboratory Animal Center of Army General Hospital approved this study (protocol no. SYXK 2014‐0037), and all experimental procedures were performed in accordance with the national guideline for animal care. All rats were housed with five rats per cage at room temperature (21‐23°C) with 60%‐70% humidity and a 12‐hour light/dark cycle. Rats had free access to standard water and rat chow.

The rat model of VAD‐induced CS was created as described previously.^7^ Briefly, female rats (n = 96) were randomly assigned to either the VAD group (n = 48), which received a modified AIN‐93G diet without any vitamin A source (Research Diets, New Brunswick, NJ) or the control group (CON, n = 48), which received an AIN‐93G diet with adequate vitamin A (4 retinol equivalents (RE)/g diet). Vitamin A deficiency was detected and confirmed in the VAD group after the rats were fed the VAD diet for more than 2 weeks as described previously.^7^, ^20^ The female rats were then mated with normal male rats at 6‐10 pm and continuously received the same diet during gestation.^21^

### 2.2 Tissue collection

Embryos were collected from six to eight pregnant rats of the CON and VAD groups on gestational days (GDs) 3, 8, 9, 11, 15 and 21.

### 2.3 Bioinformatics analysis

RNAhybrid was used to predict the miRNA‐binding sites on SULT1C2A (https://bibiserv.cebitec.uni-bielefeld.de/rnahybrid/). The targets for rno‐miR‐466c‐5p were predicted using TargetScan (www.targetscan.org/). The sequence of lncRNA SULT1C2A was downloaded from a lncRNA database (www.lncrnadb.org/), and the sequences of rno‐miR‐466c‐5p were downloaded from the miRBase (www.mirbase.org/). Information regarding protein interactions and the correlation between Foxo4 and AKT1 was obtained from the STRING database (https://string-db.org/).

### 2.4 Co‐expression network construction

A co‐expression network was constructed to identify interactions among mRNAs and lncRNAs as described previously.^19^ A weighted gene co‐expression network analysis (WGCNA) was performed to identify the associations between mRNAs and lncRNAs according to the calculated Pearson correlation coefficients. The gene encoding lncRNA SULT1C2A (length 1828 bp) is located on chromosome 9 (4621424‐4624425 [+] strand) in intron 4 of *Sult1c2a*.

### 2.5 Cell culture

HEK‐293T and H9C2 cells were maintained in high glucose DMEM (Gibco, New York, NY) added 10% foetal bovine serum (Gibco). All cells were cultured in a 5% CO_2_ humidified atmosphere at 37°C. The medium was exchanged daily, and cells were passaged every 3 days using 0.25% trypsin.

### 2.6 Cell transfection and reagents

For analysis of SULT1C2A expression, a small interfering (si)RNA against SULT1C2A (for the siRNA lnc group) and negative control oligonucleotides (for the negative control [NC] group) were synthesized by RiboBio Co., Ltd. (Guangzhou, China). For analysis of SULT1C2A‐related miRNA expression, a rno‐miR‐466c‐5p mimic (for the rno‐miR‐466c‐5p group) and scrambled oligonucleotides (for the miR‐NC group) were also synthesized by RiboBio Co., Ltd. For further analysis, the recombinant plasmids pmirGLO‐SULT1C2A‐wt1, wt2/pmirGLO‐SULT1C2A‐mt and pmirGLO‐Foxo4‐wt/pmirGLO‐Foxo4‐mt were prepared. Lipofectamine^TM^ 3000 (Invitrogen, Carlsbad, CA) was used as the transfection reagent according to the manufacturer's instructions.

### 2.7 Quantitative real‐time PCR

Total RNA was extracted from tissues using TRIzol reagent (Invitrogen, USA), according to the manufacturer's instructions. cDNAs were reverse transcribed using a First Strand cDNA Synthesis Kit (Qiagen, Germany). Then, quantitative real‐time PCR (qRT‐PCR) was performed on an ABI SYBR^®^Green PCR Master Mix (Applied Biosystems, Foster City, CA). Glyceraldehyde‐3‐Phosphate Dehydrogenase (GAPDH) was used as the reference gene. siRNA‐SULT1C2A (GCTCATGCAACCTTCCTCA) and NC‐siRNA‐SULT1C2A (TTCTCCGAACGTGTCACGT) were used to evaluate the expression ratio of SULT1C2A. For qRT‐PCR analysis of rno‐miR‐466c‐5p expression, U6 small nuclear RNA (U6 snRNA) was used as an endogenous control. In addition, melting curves were used to evaluate non‐specific amplification. The primer sequences used in this study are listed in Table 1. Relative expression levels were determined using the 2^−ΔΔCt^ method.

**Table 1:** Sequences of primers used for qRT‐PCR analysis

Gene	Primer sequence (5′‐3′)
SULT1C2A‐F	TGGCCCAGAATGAGAGGTTTGATGA
SULT1C2A‐R	CGGTGTCACAGTCCTTGGCATTTAC
Foxo4‐F	GGCGGCAAGGGTGGCAAGG
Foxo4‐R	CCGGCCTCATTGGGGACAGC
Pax1‐F	CAATGCCATCCGCCTACGAAT
Pax1‐R	AGAGACCCGCAGTTGCCTA
Nkx3‐2‐F	AGCGCCGCTTTAACCATCA
Nkx3‐2‐R	GCGTTTGGTCTTGTAGCGAC
Sox9‐F	AGTACCCGCATCTGCACAAC
Sox9‐R	ACGAAGGGTCTCTTCTCGCT
GAPDH‐F	TGGGGTGATGCTGGTGCTGAGTAT
GAPDH‐R	AGCGGAAGGGGCGGAGATGAT
rno‐miR‐466c‐5p‐F	TGTGATGTGTGTATGTAC
U6‐F	CTCGCTTCGGCAGCACA

### 2.8 Luciferase reporter assay

The target genes of SULT1C2A were predicted using a bioinformatics prediction program (miRcode and TargetScan program). PCR was performed to obtain the wild‐type SULT1C2A sequence (SULT1C2A‐wt1 and ‐wt2) and the wild‐type 3′‐UTR of *Foxo4* mRNA (Foxo4‐wt). A mutant SULT1C2A (SULT1C2A‐mt) without rno‐miR‐466c‐5p binding sites and mutant *Foxo4* 3′‐UTR (Foxo4‐mt) fragments were obtained by overlapping extension PCR with the mutant primers. The fragments, including the predicted binding sites, were cloned into a pmirGLO vector to create pmirGLO‐SULT1C2A‐wt1, pmirGLO‐SULT1C2A‐wt2 and pmirGLO‐SULT1C2A‐mt plasmids. The luciferase reporter assay was performed by cotransfection of HEK 293T cells with recombinant plasmids with rno‐miR‐466c‐5p mimics or NC plasmids using Lipofectamine^TM^ 3000. Meanwhile, H9C2 cells were transiently cotransfected with recombinant plasmids with rno‐miR‐466c‐5p mimics or siRNA lnc SULT1C2A. Luciferase activity was measured 24 hours post‐transfection with the Dual‐Luciferase Reporter Assay System (Promega, Madison, WI) according to the manufacturer's instructions. Renilla luciferase activity was normalized to firefly luciferase activity.

### 2.9 Northern blot analysis

For all miRNA samples, 20 μg of total RNA was analysed on a 17% denaturing polyacrylamide‐urea gel. mRNAs and lncRNAs were analysed on a 1.2% agarose gel in 1× MOPS solution containing 1% formaldehyde. RNAs were separated by electrophoresis and transferred onto a Hyoid‐N^+^ nylon membrane (Habersham, Freiburg, Germany). The membranes were incubated with hydration buffer containing probes in the labelling reaction system (20 mL) containing 2 mL 10× T4 PNK ligase buffer and 1 mL T4 poly nucleotide kinase (NEB, Ipswich, MA) and 2.5 mL γ‐[^32^P]‐ATP. Membranes were pre‐hybridized in the hybridization buffer at 65°C for 1 hour and hybridized overnight at 65°C. The membranes were then exposed to the phosphor imager screen for at least 12 hours.

### 2.10 Western blot analysis

For western blot analysis of protein expression, cells were lysed in radioimmunoprecipitation assay (RIPA) buffer (Sigma‐Aldrich, St. Louis, MO) containing a complete protease inhibitor cocktail (Roche, Mannheim, Germany). Proteins were separated by SDS‐PAGE and electroblotted onto polyvinylidene difluoride (PVDF) membranes. The membranes were incubated with antibodies against AKT (Affinity Biosciences, Cincinnati, OH), phosphorylated (p)‐AKT (Affinity Biosciences), PI 3 kinase p85 alpha (Abclonal, Wuhan, China) and β‐actin (Sigma‐Aldrich). Chemiluminescence was induced by the Super‐Signal West Dura Substrate (Pierce, Rockford, IL), and the images were acquired on an LAS‐3000 (Fujifilm Life Sciences, Tokyo, Japan). Staining intensity was quantified using Multi Gauge software (Fujifilm Life Sciences).

### 2.11 Statistical analysis

spss 19.0 software (SPSS, Inc, Chicago, IL) was used for all statistical analyses. Data are expressed as the mean ± SD. The Student's *t* test and one‐way ANOVA followed by Tukey's multiple comparison test were used to compare differences among samples. Correlations between SULT1C2A, *Foxo4* and rno‐miR‐466c‐5p expression were examined using Pearson's correlation analysis. *P* < 0.05 was considered statistically significant.

## 3 RESULTS

### 3.1 SULT1C2A expression is down‐regulated in embryonic tissues of rats with VAD on GD 9

Gene co‐expression networks were used to identify potential interactions among different transcripts in embryos from rats with VAD, and co‐expression modules with a high rate of protein‐coding RNAs were selected. SULT1C2A (NONRATG027649.1) was associated with many somitogenesis‐related genes, such as *Foxo4*, *Bhlha15*, *Trim55* and *Fads6*. Thus, we identified a novel lncRNA*‐*miRNA*‐*mRNA regulatory network, the SULT1C2A*‐*rno‐miR‐466c‐5p*‐Foxo4* axis in VAD.

**Figure 1:**
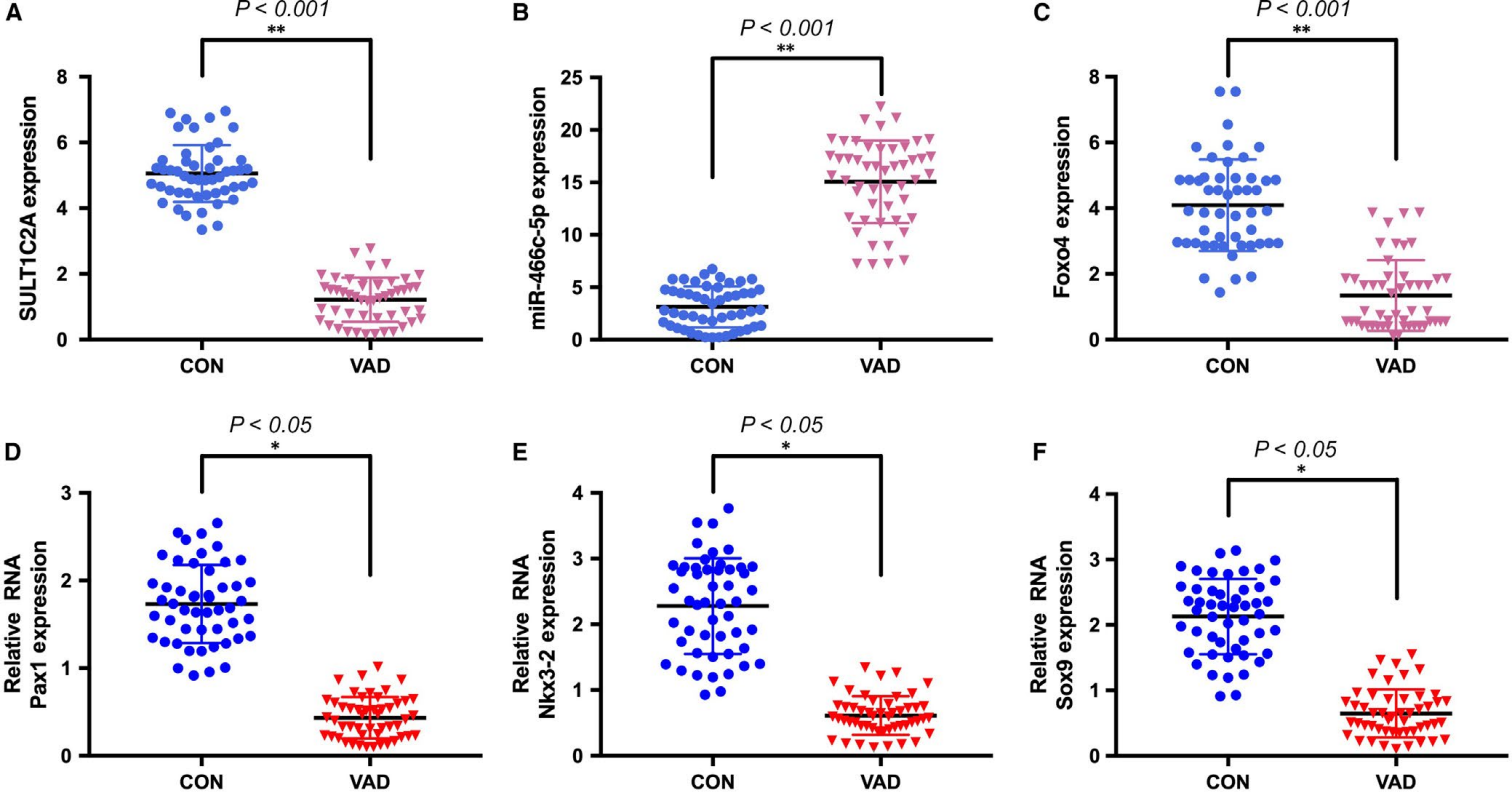
Expression of long non‐coding RNA SULT1C2A, miR‐466c‐5p and *Foxo4* in rat embryos on GD 9. (A,C) quantitative real‐time PCR results showing that relative expression levels of SULT1C2A and *Foxo4* expression were significantly lower in the vitamin A deficiency (VAD) group than in the control (CON) group. B, The relative expression level of miR‐466c‐5p was significantly increased in the VAD group compared with the CON group. (D‐F) The relative expression levels of *Pax1, Nkx3‐2* and *Sox9* were lower in rat embryos of the VAD group compared with those of the CON group. (*P < 0.05, **P < 0.001, n = 50 for each group). All experiments were independently repeated three times

We then measured the expression of SULT1C2A, rno‐miR‐466c‐5p and *Foxo4* in embryonic tissues of the VAD and CON groups on GD 9. The *Foxo4* and SULT1C2A expression levels were significantly lower in the VAD group than the CON group (Figure 1A‐C).

### 3.2 Somitogenesis is reduced in embryonic tissues of the VAD group on GD 9

We next measured the expression of somitogenesis‐associated genes in embryonic tissues from rats with VAD at GD 9. The mRNA levels of *Pax1, Nkx3‐2* and *Sox9* were significantly lower in the embryos of the VAD group compared with the CON group (Figure 1D‐F).

### 3.3 SULT1C2A directly binds to rno‐miR‐466c‐5p

**Figure 2:**
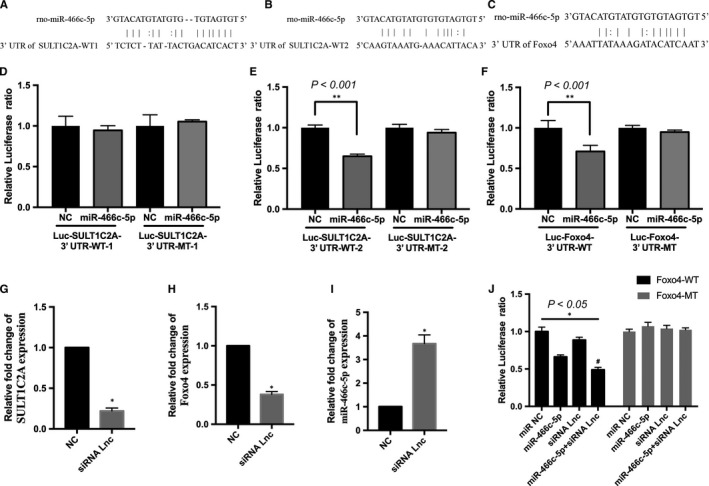
Luciferase reporter assays showed direct interaction between SULT1C2A and miR‐466c‐5p as well as between miR‐466c‐5p and *Foxo4*. (A,B) Putative binding sites between SULT1C2A‐wt1 and ‐wt2 and miR‐466c‐5p. C, Putative binding sites between *Foxo4* and miR‐466c‐5p. (D,E) Luciferase activity in cells transfected with miR‐466c‐5p mimics and pmirGLO‐SULT1C2A‐wt1, pmirGLO‐SULT1C2A‐wt2 or their mutants. rno‐miR‐466c‐5p mimics reduced the luciferase activity in cells transfected with pmirGLO‐SULT1C2A‐wt2, but not in those transfected with pmirGLO‐SULT1C2A‐wt1 or pmirGLO‐SULT1C2A‐mt. F, rno‐miR‐466c‐5p mimics reduced the luciferase activity in cells transfected with pmirGLO‐Foxo4‐wt, but not in those transfected with pmirGLO‐Foxo4‐mt. (G‐I) The relative expression levels of SULT1C2A and *Foxo4* were effectively reduced and that of rno‐miR‐466c‐5p was effectively increased in the small interfering RNA (siRNA) lnc‐transfected H9C2 cells compared with the levels in the negative control (NC)‐transfected cells. J, Treatment with the siRNA for SULT1C2A down‐regulated the expression of pmirGLO‐Foxo4‐wt but not pmirGLO‐Foxo4‐mt. (*P < 0.05, **P < 0.001, compared to the mimic NC group and ^#^ *P < *0.05, compared to the miR‐466c‐5p mimic group, n = 3). All experiments were independently repeated three times

Sequence analysis and bioinformatics analysis predicted two putative binding sites between SULT1C2A and rno‐miR‐466c‐5p (Figure 2A,B). Luciferase reporter assays showed that rno‐miR‐466c‐5p mimics reduced the luciferase activity in cells transfected with pmirGLO‐SULT1C2A‐wt2, but not with pmirGLO‐SULT1C2A‐wt1 or pmirGLO‐SULT1C2A‐mt (Figure 2D,E).

Furthermore, a siRNA against SULT1C2A was designed for transfection of H9C2 cells. According to the qRT‐PCR results, the SULT1C2A and *Foxo4* levels were effectively reduced in the H9C2 cells transfected with the siRNA Lnc compared with levels in the blank and NC groups (Figure 2G,H).

### 3.4 rno‐miR‐466c‐5p directly binds to the Foxo4 3′‐UTR

Bioinformatics analysis using TargetScan, and miRanda indicated that *Foxo4* is a potential target for rno‐miR‐466c‐5p (Figure 2C). Luciferase reporter assays showed that rno‐miR‐466c‐5p mimics reduced the luciferase activity in cells transfected with pmirGLO‐Foxo4‐wt, but not with pmirGLO‐Foxo4‐mt (Figure 2F). The qRT‐PCR results revealed that the rno‐miR‐466c‐5p level was effectively increased in H9C2 cells treated with the siRNA Lnc compared with that in the NC group (Figure 2I). In addition, transfection with rno‐miR‐466c‐5p mimics or the siRNA for SULT1C2A or cotransfection of both led to down‐regulated expression of pmirGLO‐Foxo4‐wt, but this was not observed for pmirGLO‐Foxo4‐mt (Figure 2J). These findings suggest that rno‐miR‐466c‐5p regulates *Foxo4* expression by directly binding to the 3′‐UTR and also revealed that SULT1C2A functions as a ceRNA, interacting with rno‐miR‐466c‐5p.

### 3.5 Dynamic expression of the SULT1C2A‐rno‐miR‐466c‐5p‐Foxo4 axis members in embryos

**Figure 3:**
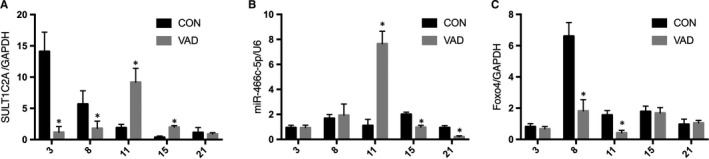
Expression profile of the SULT1C2A‐rno‐miR‐466c‐5p‐*Foxo4* axis in rat embryos on gestational days (GDs) 3, 8, 11, 15 and 21. A, The relative expression level of SULT1C2A in the vitamin A deficiency (VAD) group was significantly lower on GDs 3 and 8 and significantly higher on GDs 11 and 15. B, The relative expression level of rno‐miR‐466c‐5p was significantly increased on GD 11 but decreased on GDs 15 and 21 in the VAD group. C, Compared with that in the control (CON) group, the relative expression level of *Foxo4* in the VAD group was significantly lower on GDs 8, 11, 15 and 21. (*P < 0.05, n = 3). All experiments were independently repeated three times

We measured the expression of SULT1C2A, rno‐miR‐466c‐5p and *Foxo4* in rat embryos on GDs 3, 8, 11, 15 and 21. SULT1C2A expression in the VAD group was significantly down‐regulated on GDs 3 and 8 but up‐regulated on GDs 11 and 15 (Figure 3A). In the VAD group, rno‐miR‐466c‐5p expression was significantly up‐regulated on GD 11, but down‐regulated on GDs 15 and 21 (Figure 3B). Compared with that in the CON group, *Foxo4* expression in the VAD group was significantly down‐regulated on GDs 8 and 11 (Figure 3C).

**Figure 4:**
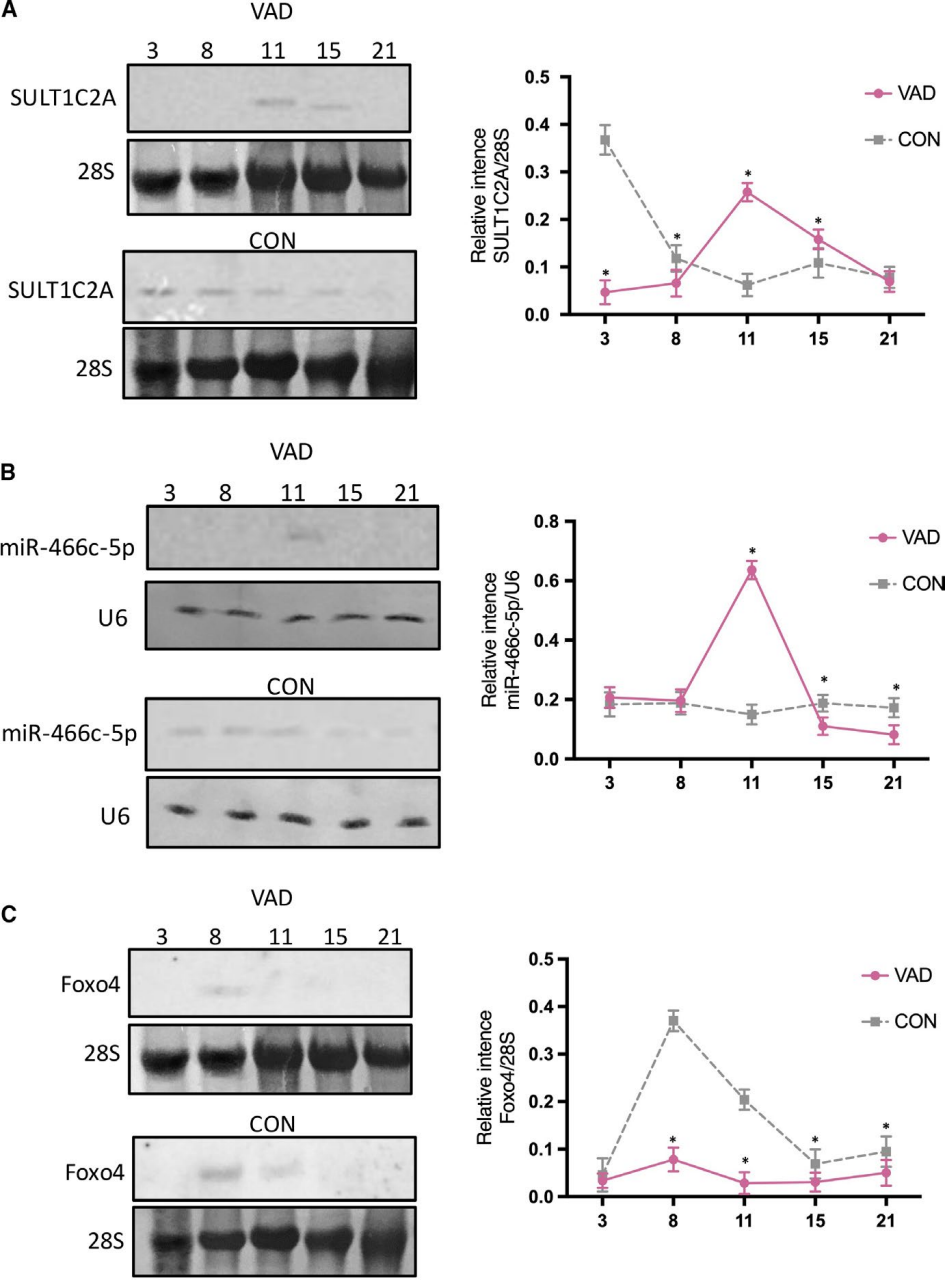
Northern blot results showing dynamic expression of the SULT1C2A‐rno‐miR‐466c‐5p‐*Foxo4* axis. A, SULT1C2A expression was significantly down‐regulated in vitamin A deficiency (VAD) rat embryos on GDs 3 and 8 but up‐regulated on GD 11. B, rno‐miR‐466c‐5p expression was significantly up‐regulated in the VAD group on GD 11 but down‐regulated on GDs 15 and 21. C, *Foxo4* expression was significantly down‐regulated on GDs 8, 11, 15 and 21. (*P < 0.05, n = 3). All experiments were independently repeated three times

Furthermore, northern blot analysis confirmed the dynamic expression of members of the SULT1C2A‐rno‐miR‐466c‐5p‐*Foxo4* axis. In the VAD group, SULT1C2A was significantly down‐regulated on GDs 3 and 8, but up‐regulated on GDs 11 and 15 (Figure 4A). Moreover, rno‐miR‐466c‐5p expression was significantly up‐regulated on GD 11, but down‐regulated on GDs 15 and 21 (Figure 4B). Finally, *Foxo4* was significantly down‐regulated on GDs 8, 11, 15 and 21 (Figure 4C).

### 3.6 PI3K‐AKT expression is altered in embryos of rats with VAD

**Figure 5:**
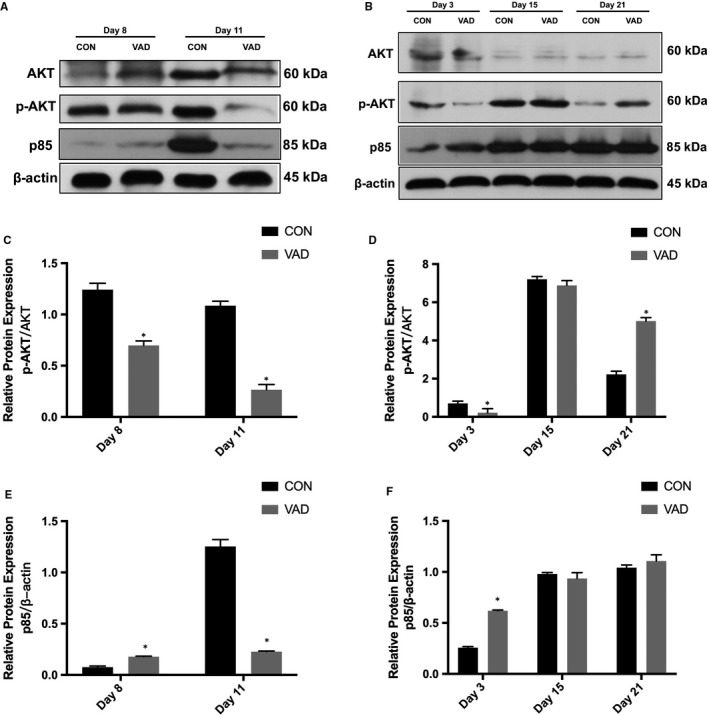
The dynamic expression of PI3K (p85), AKT and p‐AKT. A, C, E, Western blot analysis showed down‐regulation of p‐AKT in the vitamin A deficiency (VAD) group on GDs 3, 8 and 11 but up‐regulation of p‐AKT at GD 21. B, D, F, p85 expression was significantly reduced on GD 11 but increased on GDs 3 and 8 in the VAD group. (*P < 0.05, n = 3). All experiments were independently repeated three times

From the STRING database, the protein interaction and correlation score between Foxo4 and AKT1 was 0.984,^22^ and as a substrate of the PI3K‐AKT pathway, we selected this interaction as the target. The AKT, p‐AKT and PI3K (p85 regulatory subunit) expression levels were measured via western blot analysis. Compared with the CON group, AKT expression was generally up‐regulated in the VAD group, but p‐AKT expression was significantly reduced on GDs 3, 8 and 11 and then increased on GD 21 in the VAD group (Figure 5A). p85 expression was significantly reduced on GD 11 but increased on GDs 3 and 8 in the VAD group (Figure 5B). Together, these data indicate that GD 11 represents a key period of VAD during somitogenesis, which is consistent with the results of qRT‐PCT and northern blotting.

## 4 DISCUSSION

Congenital scoliosis is caused by abnormal vertebrae growth during embryogenesis, and vitamin A and RA are known to play important roles in normal physiological process. We previously found that VAD during pregnancy causes CS in postnatal rats.^7^ In the present study, we found a dynamic correlation between SULT1C2A, rno‐miR‐466c‐5p and *Foxo4* expression in embryos from a rat model of VAD‐induced CS. Our results indicated that SULT1C2A functioned as a ceRNA by directly binding to rno‐miR‐466c‐5p, and rno‐miR‐466c‐5p regulated *Foxo4* expression by directly binding to the *Foxo4* 3′‐UTR. Furthermore, the spatiotemporal expression of each gene was consistent with the dynamic expression of the SULT1C2A‐rno‐miR‐466c‐5p‐*Foxo4* axis. Our results suggest that in CS, rno‐miR‐466c‐5p down‐regulates *Foxo4* expression, and consequently reduces phosphorylation of AKT and p85 during somitogenesis on GD 11, and these effects are reversed by SULT1C2A, which competes with rno‐miR‐466c‐5p on GD 15. This investigation into the potential molecular mechanisms underlying VAD‐induced CS demonstrated dynamic changes in the expression of the SULT1C2A‐rno‐miR‐466c‐5p‐*Foxo4* axis in VAD‐induced CS, suggesting that this specific axis contributes to the pathogenesis of VAD‐induced CS.

We previously identified ceRNA regulatory networks of embryonic somite development in VAD‐induced CS and found many ncRNA expression profiles, as well as associations between mRNAs and ncRNAs in CS.^19^ Here, we further found that SULT1C2A and *Foxo4* expression levels were down‐regulated, while rno‐miR‐466c‐5p expression was up‐regulated on GD 9 in embryos of the VAD‐induced CS rat model. Furthermore, luciferase reporter assays confirmed that SULT1C2A directly targets rno‐miR‐466c‐5p and may function as a ceRNA. The assay also showed that rno‐miR‐466c‐5p inhibited *Foxo4* expression by targeting the 3′‐UTR. Our findings suggest that the lncRNA SULT1C2A plays an important role in somitogenesis in VAD‐induced CS.

Foxo4, which belongs to the family of the forkhead transcription factors, is important in embryonic development 23 and plays a key role in cartilage and skeletal muscle development.^24^, ^25^, ^26^ Foxo transcription factors regulate many cellular processes, such as apoptosis, cell cycle and differentiation. Foxo promotes growth and development of embryos by regulating the PI3K/AKT signalling pathway.^27^ It has been reported that spatiotemporal expression of *Foxo4*, which is transcriptionally regulated by PI3K signalling, is involved in the developing brain and retina in zebrafish.^28^ In addition, AKT was shown to increase Foxo4 phosphorylation in response to severe dehydration.^29^ However, we found that the dynamic expression of p85 and p‐AKT were consistent with that of *Foxo4* in embryos of the VAD group, suggesting that PI3K/AKT signalling regulates *Foxo4* expression in CS.

Retinoic acid is an important modulator that promotes embryonic cell differentiation and plays an important role in vertebrate development.^30^ Retinoic acid is required during embryogenesis as it regulates clustering of *Hox* gene expression.^31^ In addition, RA contributes to the critical role of *Foxc1a* in early somitogenesis in zebrafish.^32^ Furthermore, RA is crucial for temporal control of embryonic development, because embryonic molecular clock genes, such as limb clock *hairy2*, are regulated by RA.^33^ The PI3K/AKT pathway was also reported to play an essential role in the process of RA‐induced cell differentiation.^34^ The PI3K/AKT signalling pathway was shown to also be important for the temporal control of limb development.^33^ In MSCs, RA promotes osteogenic differentiation by activating the PI3K/AKT signalling pathway.^35^ In addition, we found abnormal changes in PI3K and AKT phosphorylation in the VAD group in the present study, suggesting that the PI3K/AKT pathway is important for VAD‐induced CS.

**Figure 6:**
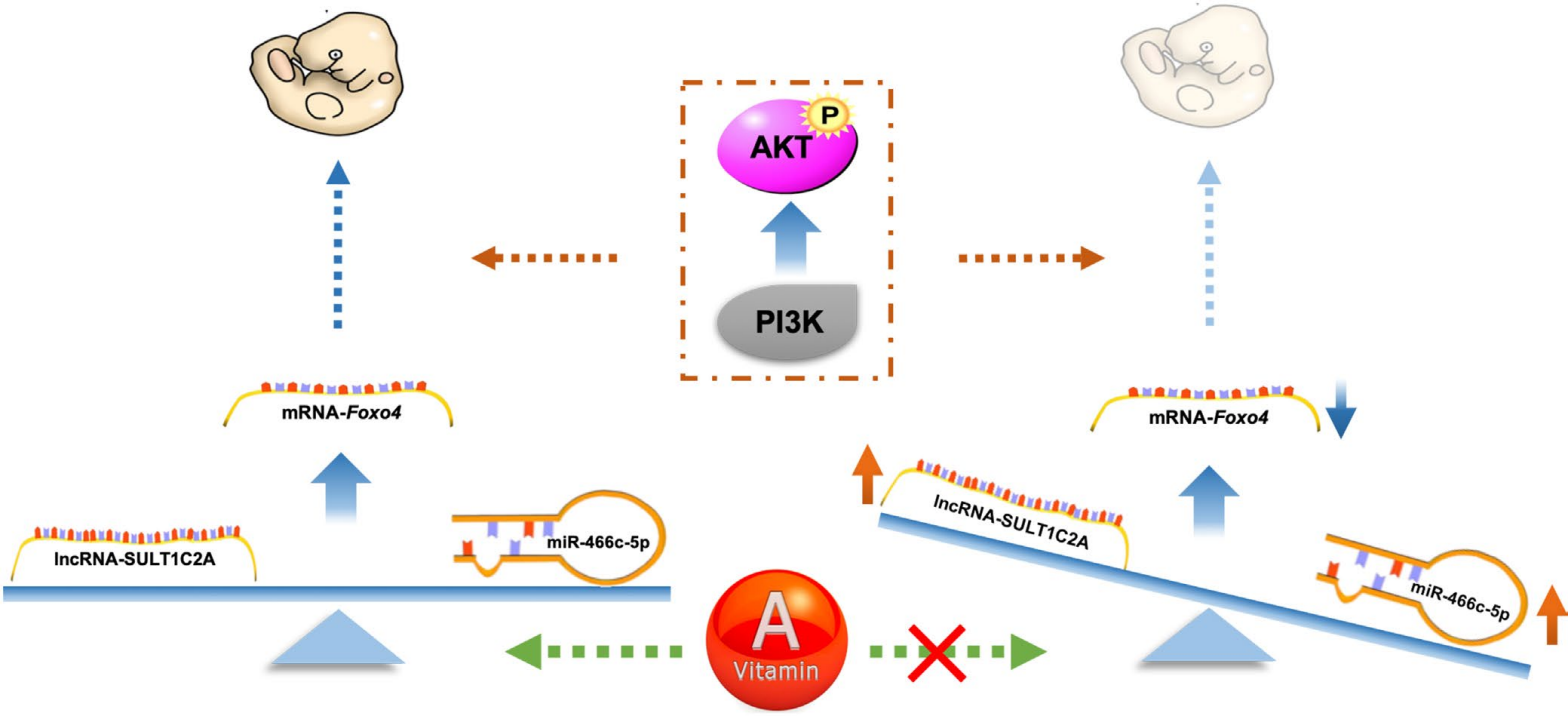
A schematic diagram of the proposed function of the lncRNA SULT1C2A‐rno‐miR‐466c‐5p‐*Foxo4* axis during somitogenesis stage from GDs 9 to 11. We speculate that VAD leads to down‐regulation of *Foxo4*, and spatiotemporally expressed lncRNA‐SULT1C2A, as a ceRNA, binds to miR‐466c‐5p to promote *Foxo4*‐associated somitogenesis of the rat embryo via activation of the PI3K‐AKT signalling pathway

Previous research indicated that the somite development of rat begins from GD 9 and ends on GD 16, with the fastest stage at GD 11 when approximately 21‐33 somites (from lower thoracic to sacral) are developed.^36^, ^37^ We specifically observed dynamic changes in the expression of the SULT1C2A‐rno‐miR‐466c‐5p‐*Foxo4* axis over GDs 8 to 15, especially from GDs 9 to 11, suggesting that the spatiotemporal changes in the expression of the SULT1C2A‐rno‐miR‐466c‐5p‐*Foxo4* axis are important for somitogenesis in VAD‐induced CS. In the VAD group, on GDs 8 and 9, lncRNA SULT1C2A and *Foxo4* expression levels were decreased but miR‐466c‐5p expression was increased on GD 9. A critical point in this balance seemed to occur on GD 11 with up‐regulation of SULT1C2A to compete with the increased miR‐466c‐5p expression. On GD 15, although *Foxo4* expression was increasing, the level was still lower than that in the CON group. Meanwhile, the expression of p‐AKT and p85 was consistent with the level of *Foxo4* expression on GD 11. Therefore, we suggest that VAD during gestation initially reduces the expression of *Foxo4*, as the target, and subsequently up‐regulates miR‐466c‐5p. To maintain the relative balance of embryonic development, lncRNA SULT1C2A is up‐regulated on GD 11 and functions as a sponge to antagonize miRNAs. The dynamic changes in the expression of the SULT1C2A‐rno‐miR‐466c‐5p‐*Foxo4* axis may be initiated on GD 9 and were most obvious on GD 11 during the early‐mid stage of somitogenesis (Figure 6). In addition, a dynamic profile of AKT phosphorylation, an important signalling pathway for somitogenesis, was found in the VAD group, and this novel finding warrants further research.

In conclusion, our results suggest that lncRNA SULT1C2A expression is increased in CS and that SULT1C2A functions as a ceRNA to spatiotemporally regulate *Foxo4* expression by targeting rno‐miR‐466c‐5p and regulating the PI3K‐ATK signalling pathway.

## CONFLICT OF INTEREST

The authors confirm that there is no conflict of interest.

## AUTHOR CONTRIBUTIONS

Chong Chen and Jianxiong Shen contributed to research conception and designed the study. Peiyu Sun, Yang Jiao, Tianhua Rong and Youxi Lin performed the experiments. Zheng Li, Jinqian Liang, Liang Sun and Lin Li analysed the data; Chong Chen, Haining Tan and Jiaqi Bi wrote the manuscript.

## Data Availability

The data that support the findings of this study are available on request from the corresponding author. The data are not publicly available because of privacy or ethical restrictions.
